# The social vulnerability index as a risk stratification tool for health disparity research in cancer patients: a scoping review

**DOI:** 10.1007/s10552-023-01683-1

**Published:** 2023-04-07

**Authors:** Tiffaney Tran, Morgan A. Rousseau, David P. Farris, Cici Bauer, Kelly C. Nelson, Hung Q. Doan

**Affiliations:** 1 Department of Dermatology, Division of Internal Medicine, The University of Texas MD Anderson Cancer Center, Houston, TX 77030 USA; 2 The University of Texas Health Science Center at Houston John P. and Kathrine G. McGovern Medical School, Houston, TX USA; 3 Research Medical Library, The University of Texas MD Anderson Cancer Center, Houston, TX USA; 4 Department of Biostatistics and Data Science, The University of Texas Health Science Center at Houston School of Public Health, Houston, TX USA

**Keywords:** Cancer, Social vulnerability, Social determinants of health, Socioeconomic factors, Neighborhood characteristics, Residence characteristics, Health inequities, Healthcare disparities, Health disparity, minority and vulnerable populations

## Abstract

**Purpose:**

The social vulnerability index (SVI), developed by the Centers for Disease Control and Prevention, is a novel composite measure encompassing multiple variables that correspond to key social determinants of health. The objective of this review was to investigate innovative applications of the SVI to oncology research and to employ the framework of the cancer care continuum to elucidate further research opportunities.

**Methods:**

A systematic search for relevant articles was performed in five databases from inception to 13 May 2022. Included studies applied the SVI to analyze outcomes in cancer patients. Study characteristics, patent populations, data sources, and outcomes were extracted from each article. This review followed the Preferred Reporting Items for Systematic Reviews and Meta-Analyses (PRISMA) guidelines.

**Results:**

In total, 31 studies were included. Along the cancer care continuum, five applied the SVI to examine geographic disparities in potentially cancer-causing exposures; seven in cancer diagnosis; fourteen in cancer treatment; nine in treatment recovery; one in survivorship care; and two in end-of-life care. Fifteen examined disparities in mortality.

**Conclusion:**

In highlighting place-based disparities in patient outcomes, the SVI represents a promising tool for future oncology research. As a reliable geocoded dataset, the SVI may inform the development and implementation of targeted interventions to prevent cancer morbidity and mortality at the neighborhood level.

**Supplementary Information:**

The online version contains supplementary material available at 10.1007/s10552-023-01683-1.

## Introduction

The incidence of cancer in the U.S. is projected to rise in part due to increased diagnosis in aging populations and minority groups [1]. With this rise in incidence, the annual national costs for cancer-related medical services and treatments is projected to swell from $185 billion in 2015 to $246 billion by 2030 [2]. Social determinants of health (SDOH), which are non-medical factors such as socioeconomic status (SES), race, and ethnicity that influence health outcomes, are known to contribute to disparities in cancer incidence and mortality [3–7]. Identifying and understanding health disparities in cancer patients can inform initiatives designed to prevent excess cancer morbidity and mortality, decrease economic costs to society, and promote health equity.

**Fig. 1:**
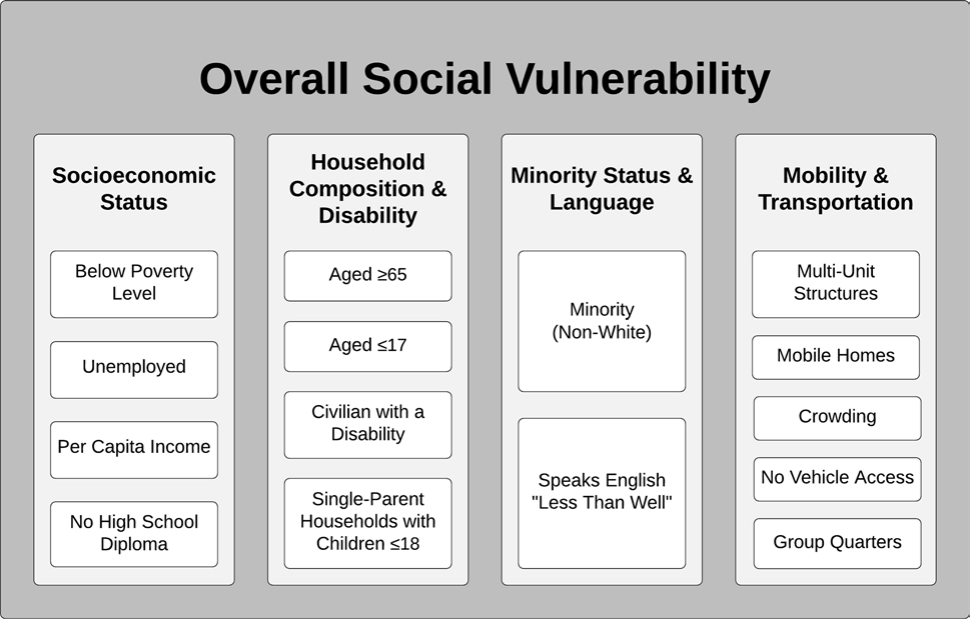
Social variables captured by the CDC/ATSDR SVI, categorized into four subthemes

Intensive effort has been devoted to the collection of objective data measuring important social variables that impact health and quality of life. Examples of publicly accessible metrics include the Community Need Index [8], the Area Deprivation Index [9], the Distressed Communities Index [10], and the novel Social Vulnerability Index (SVI) [11]. Examples of SES-based metrics developed specifically for cancer registry data analyses include the Yost index [12] and the Yang index [13]. The SVI generally differs from these other indices in that it contains 15 different social variables and offers granular data for U.S. administrative units at the county and census tract-level (**Fig. ****1**). Its multiple dimensions offer rich potential for comprehensive assessment of how SDOH may impact cancer care across broad geographic areas.

Developed by the Centers for Disease Control and Prevention (CDC), the SVI was originally created to guide the allocation of government resources to vulnerable communities in the event of a natural or man-made disaster or a disease outbreak [11]. Vulnerable populations are defined as those who have special needs, and in the context of healthcare, these may include persons with a low SES, without a vehicle for transportation, and/or with limited English proficiency [11]. As a validated tool for disaster preparedness, recovery, and adaptation decisions [14], the SVI does not capture all SDOH such as insurance status, but it offers rich insight into many SDOH that are relevant to patient outcomes. It organizes data from the ACS into the subthemes of SES, household composition, minority status, and housing. Recently, the SVI was utilized in the assessment of SDOH on morbidity and mortality from the SARS-CoV-2 virus (COVID-19) and COVID-19 vaccine coverage [15, 16].

Previous studies have examined how various SDOH impact patients along the cancer care continuum, revealing greater cancer burden in vulnerable populations [17]. This longitudinal framework outlines cancer control areas beginning with primary prevention (e.g., exposures to risk factors) and secondary prevention (e.g., early detection, screening) and continuing onto diagnosis, treatment, survivorship care, and end-of-life care [18]. Health disparities due to social factors and neighborhood characteristics seem to impact nearly every aspect of this continuum from incidence to end-of-life [19–22]. The objective of this study was to explore innovative applications of the SVI to current oncology research through a literature review and to identify further research opportunities using the cancer care continuum framework.

## Methods

This review was conducted in accordance with the Preferred Reporting Items for Systematic Reviews and Meta-Analyses (PRISMA) guidelines [23].

### Eligibility criteria

The inclusion and exclusion criteria for study eligibility are listed in Supplementary Table S1. Studies deemed eligible for inclusion used the CDC SVI to measure any primary or secondary outcomes in the population of interest. No limiters were implemented in terms of specific data sources or outcome measures. The population of interest comprised patients identified as at risk for cancer, diagnosed with cancer, undergoing a therapeutic intervention for a cancer diagnosis, or under longitudinal surveillance for cancer recurrence.

### Data sources

A systematic search for relevant articles was performed in MEDLINE (Ovid), Embase (Ovid), Web of Science Core Collection (Clarivate), Scopus (Elsevier), and PubMed (National Library of Medicine) from database inception to 13 May 2022. A medical research librarian (D.P.F.) developed and tailored the search strategy to each database. Controlled vocabulary (MeSH and Emtree) and natural language terms were selected for the concepts of *social vulnerability* and *cancer*. The full search strategy for each database is included in Supplementary Methods S1. Searches were limited to articles published in the English language, but no other limiters or published search filters were used. Gray literature resources including conference abstracts were included as an additional source for relevant articles.

EndNote X9 (Clarivate) was used to remove duplicate results. Deduplicated results were then uploaded to Rayyan, a free web application developed to facilitate collaborative systematic reviews.

### Study selection

Three investigators (T.T., H.Q.D., and M.A.R.) independently screened the titles and abstracts of identified records using Rayyan. All records deemed potentially relevant by at least one investigator were retrieved for full-text review. Three investigators (T.T., H.Q.D., and M.A.R.) then independently assessed all full-text manuscripts for study eligibility and voted on their inclusion or exclusion. The classification of each article was based on a unanimous vote. For articles in disagreement, input from a fourth investigator (K.C.N.) was then solicited and incorporated into a decision-making process based on group consensus. Overlapping samples were also identified and resolved. In the circumstance that identical samples and analyses were presented in both a conference abstract and a journal article, the peer-reviewed publication was selected for inclusion. The reference lists of included articles were also examined to identify any additional relevant articles. The study selection process is summarized in the PRISMA flow diagram (Supplementary Figure S1).

### Data extraction

A group of three investigators (T.T., M.A.R., and H.Q.D.) performed the data extraction process in which each included article was assigned to two of the three investigators for close review and data extraction. The following variables were extracted: study design, research question, data sources, patient population, use of the SVI, other SDOH measures, primary outcomes, secondary outcomes, and effect measures. Any discordance was resolved by group consensus with the supervision of a fourth investigator (K.C.N.).

### Reporting quality assessment

Three investigators (T.T., M.A.R., and H.Q.D.) assessed the reporting quality of the included articles using a scoring worksheet adapted for the purposes of this review (Supplementary Methods S2). Developed for the appraisal of observational studies [24], the worksheet reflects the 22 items on the Strengthening the Reporting of Observational Studies in Epidemiology (STROBE) checklist [25].

For each item on the checklist, studies received one point for complete reporting, half a point for incomplete reporting, or zero points for no reporting. Based on the total average number of points, the quality of each study was classified as excellent (full 22 points), good (19–21 points), fair (14–18 points), and low (0–13 points). The reporting quality of each article was assessed and scored by two out of three investigators (T.T., M.A.R., and H.Q.D.), and the average of the individual scores determined the final quality classification. To receive a high score, studies must have had described efforts to address potential sources of bias, control for cofounding factors, and provide unadjusted and adjusted estimates [25].

## Results

A total of 593 results were retrieved from the five databases (MEDLINE, *n* = 81; Embase, *n* = 160; Web of Science, *n* = 132; Scopus, *n* = 117; PubMed, *n* = 103). Following deduplication, 260 unique records were identified. Of the 73 full-text manuscripts assessed for study eligibility, 31 met all criteria for inclusion [26–56]. A summary of the 31 included studies is presented in Table 1, and their primary and secondary outcomes are shown in Supplementary Table S2. Of the 31 included studies, 28 (90.3%) executed a cross-sectional study design based on secondary data analyses [26–30, 32–41, 43–45, 47–56], and 3 (9.7%) studies executed a retrospective cohort study design with longitudinal follow-up of patient outcomes [31, 42, 46].

**Table 1:** Summary of articles included in this review (*n* = 31)

Article ^[Refs.]^ (Author, Year, *Journal*)	Cancer types (No. patients)	Research question	Patient data sources, years	Cancer care continuum	Reporting quality
Abbas et al., 2021, *Ann Surg Oncol *[26]	Colon (*n* = 26,287) Lung (*n* = 16,645) Pancreatic (*n* = 6,183) Rectal (*n* = 3,174) Esophageal (*n* = 1,427)	Are county-level SVI and race/ethnicity associated with patterns of hospice utilization among those who underwent a cancer resection and who lived ≥ 30 days after the surgery?	Medicare, 2013–17	End-of-life	Excellent
Azap et al., 2020, *Surgery* [27]	Pancreatic (*n* = 18,841) Liver (*n* = 13,301)	Is county-level SVI associated with the probability of post-op textbook outcomes in those who underwent a cancer resection?	Medicare, 2013–17	Treatment Recovery	Excellent
Azap et al., 2021, *Ann Surg Oncol* [28]	Pancreatic (*n* = 15,931)	Are county-level SVI and race/ethnicity associated with the probability of undergoing a cancer resection or receiving chemotherapy?	SEER-Medicare, 2004–16	Treatment	Excellent
Azap et al., 2021, *JAMA Surg* [29]	Liver (*n* = 10,888)	Are county-level SVI and race/ethnicity associated with the probability of undergoing a cancer resection or transplant?	SEER-Medicare, 2004–17	Treatment	Excellent
Barmash et al., 2020, *J Am Coll Surg* [30]	Colon (*n* ~ 35,324)	Is county-level SVI associated with emergent vs. non-emergent colon resection and post-op outcomes?	Medicare, 2016–17	Treatment Treatment Recovery	Low
Bhandari et al., 2021, *Blood* [31]	Acute myeloid leukemia (*n* = 652 in California)	Is census tract-level SVI associated with 1-year non-relapse mortality?	Single institution(s) in California, 2013–19	N/A	Low
Bowers et al., 2020, *Lab Invest* [32]	Leukemia (NR; 727 counties in 14 states)	Does leukemia subtype incidence vary within and between areas of SES disparity? *SDOH Measures*: Gini, income inequality, county-level SVI, residential segregation, rurality	SEER-21, 2000–16	Diagnosis	Low
Carmichael et al., 2022, *Am J Surg* [33]	Colorectal (*n* = 392)	Is census tract-level SVI associated with increased risk of post-op 30-day morbidity following colectomy?	Single institutions in Colorado, 2013–2017	Treatment Recovery	Good
Dalmacy et al., 2021, *Surgery* [34]	Pancreatic (NR) Liver (NR)	Is county-level SVI associated with risk of fragmented post-op care among those who had ≥ 1 readmission within 90 days?	Medicare, 2013–17	Treatment Treatment Recovery	Excellent
Diaz et al., 2021, *Ann Surg Oncol* [35]	Lung (*n* = 14,403 in California) Rectal (*n* = 7,520 in CA) Pancreatic (*n* = 3,744 in CA) Esophageal (*n* = 1,270 in CA)	Is county-level SVI associated with utilization of high-volume hospitals for high-risk cancer operations?	California OSHPD, 2012–16	Treatment	Excellent
Diaz et al., 2021, *J Gastrointest Surg* [36]	Colon (*n* = 33,312)	Is county-level SVI associated with the probability of having a non-elective vs. elective colon resection?	MedPAR, 2016–17	Treatment Treatment Recovery	Excellent
Diaz et al., 2021, *J Surg Oncol* [37]	Lung (*n* = 33,803) Colon (*n* = 21,939)	What is the impact of county-level SVI and racial/ethnic residential diversity on post-op outcomes among those who underwent a cancer resection?	Medicare, 2016–17	Treatment Treatment Recovery	Excellent
Diaz et al., 2021, *Surgery* [38]	Pancreatic (*n* = 13,393) Liver (*n* = 3,594)	Is county-level SVI associated with use of a high-volume or Magnet recognition hospital?	Medicare, 2013–17	Treatment Treatment Recovery	Excellent
Ganatra et al., 2021, *Circulation* [39]	Any (NR)	Is county-level SVI associated with mortality from cardiovascular disease and concomitant cancer (cardio-oncology)?	CDC WONDER, 2014–18	N/A	Low
Grant et al., 2021, *Blood* [40]	Liquid tumors (e.g., multiple myeloma, leukemia, lymphoma) (*n* = 338 in North Carolina)	Is county-level SVI associated with physical frailty?	Carolina Senior Registry, NR Registry for Adults with PCDs, NR	N/A	Low
Grant et al., 2021, *J Clin Oncol* [41]	Multiple myeloma (NR; 456 trials registered in North Carolina)	Is county-level SVI associated with multiple myeloma trial availability in North Carolina?	ClinicalTrials.gov, accessed 01/24/2021	Treatment	Low
Hawley et al., 2022 *JAMA Netw Open* [42]	Any, except NMSCs (*n* = 4,749)	Is there a spatiotemporal association in COVID-19 outcomes?	CCC19, Mar.–Nov. 2020	Treatment	Excellent
Hyer et al., 2021, *J Am Coll Surg* [43]	Colon (*n* = 113,929) Lung (*n* = 70,642) Rectal (*n* = 14,849) Esophageal (*n* = 4,380)	Are there differences in textbook outcomes relative to county-level SVI and race/ethnicity among those who underwent a cancer operation?	Medicare, 2013–17	Treatment Treatment Recovery	Excellent
Labiner et al., 2022, *J Gastrointest Surg* [44]	Pancreas? Liver? (*n* = 26,540)	Does county-level SVI subtheme analysis in patients undergoing hepatopancreatic surgery better stratify potential gaps in identifying risks of post-op complications?	Medicare, 2013–17	Treatment Recovery	Good
McAlarnen et al., 2021, G*ynecol Oncol* [45]	Ovarian, uterine, cervical, or vaginal/vulvar (*n* = 270 in NYC)	Is census tract-level SVI associated with the utilization of virtual gynecologic oncology visits at the beginning of the COVID-19 pandemic?	Single institution(s) in NYC, 2020	Survivorship Care	Good
McAlarnen et al., 2022, *Cancer Epidemiol Biomark Prev* [46]	Cervical (*n* = 66 in Milwaukee, Wisconsin)	Are demographic and geographic factors (e.g., census tract-level SVI) associated with diagnosis of locally advanced cervical cancer?	Single institution(s) in Milwaukee, Wisconsin, 2016–21	Diagnosis	Low
Mock et al., 2021, *Transplant Cell Ther* [47]	Acute myeloid leukemia (*n* = 818 in Virginia)	Do hematopoietic cell transplantation rates for acute myeloid leukemia vary between different regions in Virginia? Or vary by census tract-level SVI?	Virginia Cancer Registry, 2013–17	Treatment	Excellent
Pan et al., 2021, *Hepatology* [48]	Liver (*n* = 99 in NYC)	Do neighborhood-level (e.g., census tract-level) characteristics impact alcohol-associated liver disease outcomes (including development of HCC)?	Single institution(s) in NYC, 2012–19	Prevention Diagnosis	Low
Papageorge et al., 2021, *J Am Coll Surg* [49]	Liver (*n* = 19,751 in 12 states)	Was county-level SVI associated with the impact of Medicaid expansion on the diagnosis of HCC?	SEER, 2010–16	Diagnosis	Excellent
Parks et al., 2022, *JAMA* [50]	Any (*n* = 3,619,393 in 1,206 counties)	Is county-level tropical cyclone exposure associated with county-level cause-specific mortality (including cancer-specific mortality)?	CDC NCHS, 1988–18	Prevention	Excellent
Puvvula et al., 2021, *Water* [51]	All pediatric cancers (*n* = 2,559 in Nebraska)	Are atrazine concentrations at watersheds associated with the incidence of pediatric cancers in Nebraska? *Model*: adjusted for census tract-level SVI	Nebraska Cancer Registry, 1987–16	Prevention Diagnosis	Good
Rice et al., 2021, *Ann Surg Oncol* [52]	Liver (*n* = 14,369)	What factors (e.g., county-level SVI) are associated with hospice utilization and end-of-life healthcare expenditures among those who died of HCC?	SEER-Medicare, 2004–16	End-of-life	Excellent
Taylor et al., 2021, *Gastroenterology* [53]	Colon (*n* = 146,041)	Is Medicaid-Medicare dual eligibility status associated with non-elective surgery for colon cancer and variation in outcomes and spending? *Analyses*: stratified by county-level SVI	Medicare, 2014–18	Treatment	Low
Ying et al., 2020, *Hepatology* [54]	Liver (*n* = 143 in NYC)	Are racial and neighborhood-level factors (e.g., SVI) associated with adverse outcomes in those with cirrhosis due to viral hepatitis?	Single institutions(s) in NYC, 2012–19	Prevention Diagnosis	Low
Ying et al., 2021, *Hepatology* [55]	Liver (*n* = 98 in NYC)	Is SVI associated with advanced-stage HCC diagnosis in those with cirrhosis due to viral hepatitis?	Single institution(s) in NYC, 2012–20	Prevention Diagnosis	Low
Zhang et al., 2022, *Health Aff (Project Hope)* [56]	Colon (*n* = 203,732)	Is county-level SVI associated with unplanned surgeries for one of three specified access-sensitive conditions (including colectomy for colon cancer)?	MedPAR, 2014–18	Treatment	Excellent

^*^Nationwide, unless otherwise indicated

*SVI* social vulnerability index, *post-op* post-operative, *SEER* Surveillance, Epidemiology, and End Results Program, *SDOH* social determinants of health, *SES* socioeconomic status, *CA* California, *OSHPD* Office of State-wide Health Planning and Development, *MedPAR* Medicare Provider Analysis and Review, *CDC* Centers for Disease Control and Prevention, *WONDER* Wide-ranging Online Data for Epidemiologic Research, *PCD* plasma cell disorder, *NMSC* non-melanoma skin cancer, *COVID-19* coronavirus disease 2019, *CCC19* COVID-19 and Cancer Consortium, *NYC* New York City, *HCC* hepatocellular carcinoma, *NCHS* National Center for Health Statistics, *NR* not reported

### Reporting quality

We appraised the reporting quality of each study, placing emphasis on potential biases and confounding factors, and these results are summarized in Table 2. A majority (51.6%) of studies were rated as demonstrating excellent reporting quality [26–29, 34–38, 42, 43, 47, 49, 50, 52, 56]. Overall, these studies were more likely to address internal validity by describing potential biases and confounders (Supplementary Methods S2). Of the 11 studies judged to be of poor reporting quality, all were conference abstracts [30–32, 39–41, 46, 48, 53–55], most of which did not include descriptions of their statistical methods or strategies to mitigate biases or adjust for confounders. Conference abstracts typically face strict space limitations and do not undergo the same rigorous peer review as journal publications. Nevertheless, they were included given the recent introduction of SVI-based population analyses in the literature and the relative paucity of relevant articles in oncology research.

**Table 2:** Distribution of included articles by reporting quality category

Reporting Quality Category	STROBE Score Range (0–22)	No. Articles [Refs.] (%)
*Excellent* These articles had no missing elements and accounted for potential biases and confounding factors	22 21 if only missing declaration of funding source	16 [26–29, 34–38, 42, 43, 47, 49, 50, 52, 56] (51.6%)
*Good* These articles had only a few missing elements	19–21	4 [33, 44, 45, 51] (12.9%)
*Fair* These articles had many missing elements	18–14	0 (0.0%)
*Low* These articles had limited reporting. Meeting abstracts generally fall in this category	0–13	11 [30–32, 39–41, 46, 48, 53–55] (35.5%)

*STROBE* Strengthening the Reporting of Observational Studies in Epidemiology

Reference: von Elm E, et al. The Strengthening the Reporting of Observational Studies in Epidemiology (STROBE) statement: guidelines for reporting observational studies. *J Clin Epidemiol*. Apr 2008;61(4):344–9

### Patient populations

This review encompassed patients identified as at risk for cancer, diagnosed with cancer, undergoing cancer treatment, or receiving cancer survivorship care. The distribution of included articles by cancer type is summarized in Table 3. Ten studies addressed liver cancer [27, 29, 34, 38, 44, 48, 49, 52, 54, 55], and eight studies addressed colon or colorectal cancer [26, 30, 33, 36, 37, 43, 53, 56]. Other malignancies examined by multiple studies included pancreatic [26, 28, 34, 35, 38, 44], lung [26, 35, 37, 43], rectal [26, 33, 35, 43], and esophageal cancer [26, 35, 43].

**Table 3:** Distribution of included articles by cancer type

Cancer Type	No. Articles [Refs.]
*Solid tumors*	
Bladder cancer	–
Brain cancer	–
Breast cancer	–
Cervical cancer	2 [45, 46]
Colon cancer	8 [26, 30, 33, 36, 37, 43, 53, 56]
Esophageal cancer	3 [26, 35, 43]
Kidney cancer	–
Liver cancer	10 [27, 29, 34, 38, 44, 48, 49, 52, 54, 55]
Lung cancer	4 [26, 35, 37, 43]
Melanoma	–
Ovarian cancer	–
Pancreatic cancer	6 [26, 28, 34, 35, 38, 44]
Prostate cancer	–
Rectal cancer	4 [26, 33, 35, 43]
Skin cancer, non-melanoma	–
Stomach cancer	–
Thyroid cancer	–
Uterine cancer	1 [45]
Vaginal/vulvar cancer	1 [45]
*Liquid tumors*	
Leukemia (including AML)	3 [31, 32, 40]
Lymphoma	1 [40]
Myeloma (including MM)	2 [40, 41]
Unspecified liquid tumors	2 [31, 40]
Any cancers	2 [39, 50]
Any cancers, excluding NMSC	1 [42]
Any pediatric cancers, excluding NMSC	1 [51]

*HCC* hepatocellular carcinoma, *PDAC* pancreatic adenocarcinoma, *AML* acute myeloid leukemia, *MM* multiple myeloma, *NMSC* non-melanoma skin cancer

### Patient data sources

For studies performed across all states, common sources for patient data were the Centers for Medicare & Medicaid Services (CMS) [30] and the Surveillance, Epidemiology, and End Results (SEER) Program [49]. Specific datasets included the Medicare Single Analytic Files [26, 27, 34, 37, 38, 43, 44, 53], Medicare Provider Analysis and Review [36, 56], SEER-21 [32], and the SEER-Medicare linked database [28, 29, 52]. Other specific national data sources included the National Center for Health Statistics [50], the CDC Wide-ranging ONline Data for Epidemiologic Research database [39], the COVID-19 and Cancer Consortium Database [42]. In addition, one study utilized data obtained from a national clinical trials database (ClinicalTrials.gov) [41]. For studies performed in single states, data sources included state-wide health databases [35], senior registry databases [40], and cancer registry databases [40, 47, 51]. In addition, seven studies used de-identified patient data from single institutional electronic medical records [31, 33, 45, 46, 48, 54, 55].

Overall, many of the included studies were population-based cross-sectional studies with minimal risk for selection bias. Many employed retrospective data from national or state registry databases, administrative billing data, or institutional medical records and included all eligible patients (e.g., patients with the diagnosis of interest) in the analytic cohorts.

### Use of SVI data

The SVI database provides data at the county or census tract-level [11]. Each residential address in the U.S. has a unique 15-digit geographic identifier (GEOID) [57]. Created by the Census Bureau, each GEOID consists of a 2-digit state code, 3-digit county code, 5-digit census tract code, and/or 4-digit census block code [57]. In the U.S., the relationship between these geographic entities is such that states are subdivided into counties which are further subdivided into census tracts. The GEOID may thus reflect a patient’s county or census tract, and this numeric string can then be used to link patient data with area-level SVI data. In most cases, a patient’s county of residence can be deduced from their ZIP Code™ data, whereas identifying a patient’s census tract of residence typically requires collection of their full postal address data. A majority (67.7%) of studies in this review performed analyses at the county level [26–30, 32, 34–43, 49, 50, 52, 53, 56]. Seven studies conducted analyses at the census tract-level [31, 33, 45–48, 51].

The statistical treatment of SVI data also varied across studies. For each variable and subtheme, the SVI database provides percentile rankings ranging from 0 to 100% (least to greatest social disadvantage) [11]. Depending on the specific dataset, counties or census tracts are ranked against each other across a single state or across all states [11]. A patient residence’s SVI value may vary based on the geographic unit of analysis and the comparator group (e.g., all counties in a single state, all census tracts across all states). Some studies analyzed patient outcomes using the overall SVI percentile ranking for the patient residential address [31, 34, 35, 47, 48, 51]. Others used the percentile ranking for subthemes [31, 34, 44], percentile ranking for specific variables [49], median overall SVI [45, 46], and median subtheme SVI [46]. Some studies used 1-unit [41], 10-unit [26, 28], or 20-unit [30] references to calculate odds ratios, and a few treated SVI as a continuous variable [42, 52, 56]. A majority (52.4%) of studies stratified patients’ respective geographic units into SVI quartiles [27–29, 32, 33, 35–39, 43, 52–54]. While the quartiles did not necessarily align with the overall SVI percentile rankings, they offered insight into the SVI distribution across a patient population. Other studies stratified by tertiles [31, 50], quintiles [40, 56], or deciles [56].

### Other SDOH measures

Eight studies investigated neighborhood characteristics using other SDOH measures in addition to the SVI [32, 37, 45, 47–49, 54, 55]. Area-level SDOH based on county or ZIP Code™ data included race and ethnicity [45], foreign-born status [48, 54, 55], median household income [37, 45], educational attainment [45], limited English proficiency [37, 45, 47], employment rate [55], poverty rate [47], insurance status [55], rurality [32, 42, 47], and neighborhood walk score [54, 55]. One study applied multiple county-level metrics such as the Gini coefficient [58], income inequality ratio, and residential segregation in addition to the SVI [32]. Another study integrated county-level variables from the Behavioral Risk Factor Surveillance System such as obesity, tobacco use, and alcohol use [49]. Some studies examined individual-level SDOH beyond race/ethnicity such as educational attainment [40], employment [45], marital status [29, 45, 47], insurance status [35, 50, 53], and rurality [47].

### Outcome measures

Many studies in this review addressed multiple components of the cancer care continuum. The distribution of included articles along the cancer care continuum is summarized in Supplementary Table S3. Along the continuum, five studies applied the SVI to examine geographic disparities in potentially cancer-causing exposures (e.g., oncogenic viruses, environmental contaminants or characteristics) [42, 48, 50, 51, 54, 55]; seven to examine disparities in cancer diagnosis (e.g., all-stage, early stage, late stage) [32, 46, 48, 49, 51, 54, 55]; fourteen in cancer treatment (e.g., surgery receipt, chemotherapy receipt, stem cell transplantation, clinical trial access) [27–30, 34–38, 41–43, 47, 53, 56]; nine in treatment recovery (e.g., post-operative complications, readmission rates) [27, 30, 33, 34, 36–38, 43, 44]; one in survivorship care [45]; and two in end-of-life care [26, 52]. Fifteen (48.4%) studies also examined disparities in mortality using the SVI [27, 30, 31, 33, 36–39, 42–44, 48, 50, 54, 55]. Studies that examined disparities in secondary prevention (e.g., early detection, screening) were not identified.

### Limitations

We assessed the limitations of each study. A large majority of the included studies used a cross-sectional study design in which samples represented a single timepoint. These studies may not capture changes in residence among cancer patients over the course of their disease. For instance, patients may relocate to a different county with a different level of social vulnerability as they seek to live with or near caregivers, improve perceived access to healthcare, receive advanced cancer treatment, or explore a new lifestyle. Thus, the patient residence’s SVI at time of cancer diagnosis may significantly differ from that at time of cancer treatment or end-of-life care.

For the studies that relied on Medicare administrative billing data, a major limitation was low generalizability to younger patient populations since the Medicare population comprises patients aged 65 or older [26, 27, 30, 34, 36–38, 43, 44, 53, 56]. The Medicare population also excludes those aged 65 or older without insurance or with private insurance. When compared to the general population, White patients tend to be over-represented in the Medicare dataset, while non-White minority patients are under-represented. Administrative billing data also lacked relevant patient clinical information (e.g., stage at diagnosis, medical comorbidities, referral patterns, refusal of treatment/care) and individual-level SDOH information (e.g., education, income, employment) [29, 35, 47]. In addition, studies based on administrative billing or disease registry data may be subject to information bias from miscoding, incomplete coding, noncoding, or under-ascertainment. Incomplete or inaccurate residential address data could also affect the collection and treatment of area-level SVI data.

For studies that performed analyses at the county level [26–30, 32, 34–38, 41, 43, 49, 52, 54, 55], results may not be applicable to specific census tracts or blocks across the county given potential heterogeneity within the county, especially in urban areas. Similarly, area-level results, whether county- or census tract-level, may not be generalizable to individual patients. Studies that primarily relied on data from a single state [35, 47, 51] or single medical center [45, 48, 54, 55] also had limited generalizability to other states or institutions, respectively.

## Discussion

This review seeks to demonstrate the potential utility of the SVI, a composite scale encompassing many different SDOH, as applied to oncology research. Given the variability in reporting quality among the included studies, the diversity of patient populations represented, and the wide range of outcome measures examined, a meta-analysis was precluded in this sutdy. The results of this study rather provide an overview of the wide range of studies related to the SVI that can be found in the indexed oncology literature. As more higher quality studies with rigorous methodologies and analyses are published, future research may apply statistical methods to synthesize findings from studies with similar patient populations and outcome measures.

To describe the current literature, we employed the cancer care continuum as an evaluation framework. In oncology research, the cancer care continuum helps identify research and policy needs to prevent excess cancer morbidity and mortality. In regard to disparities in cancer treatment, recovery, and survivorship, studies in this review demonstrated that with increasing SVI, cancer patients were less likely to receive neoadjuvant chemotherapy [28], less likely to utilize a high-volume hospital for surgical resection [35, 38], more likely to experience post-operative complications [36, 37], less likely to achieve post-operative textbook outcomes [27, 43], and more likely to encounter fragmented post-operative care [34]. With respect to race and ethnicity, studies in this review showed that minority patients also had a lower likelihood than White patients of receiving neoadjuvant chemotherapy [28] or achieving post-operative textbook outcomes as SVI increased [43]. These studies demonstrate how certain vulnerable populations experience worse outcomes in part due to social factors that could potentially be mitigated with geographically targeted interventions.

**Fig. 2:**
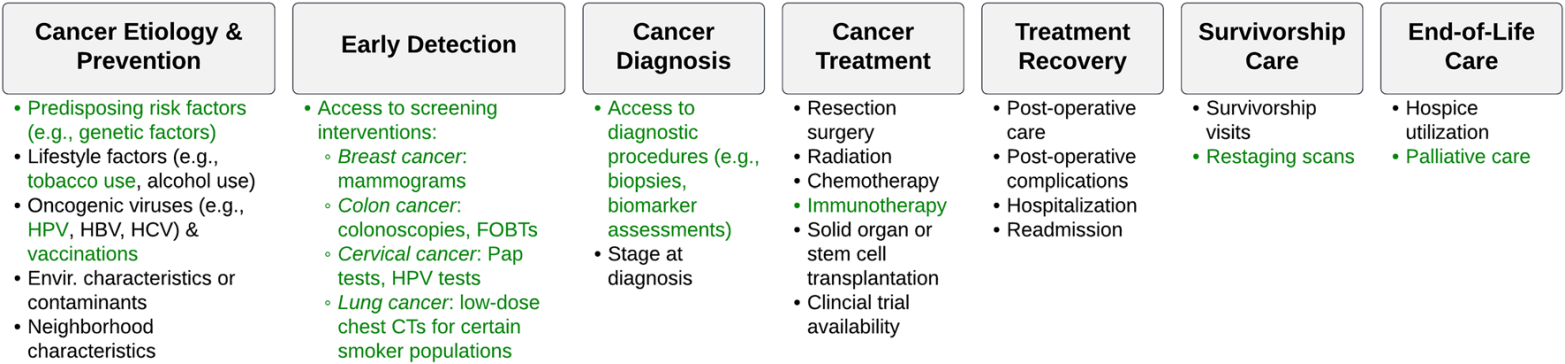
Distribution of literature findings (in black) and research opportunities (in **green**) along the cancer care continuum

A depiction of future opportunities for health disparities research using the SVI can be found in Table 3 and Fig. 2 with respect to cancer types and components of the cancer care continuum, respectively. For example, the SVI could be applied to examine disparities in the socioeconomic and geographic coverage of early detection initiatives such as cancer screening campaigns (e.g., mammograms, Papanicolaou tests, colonoscopies) [59]. To date, many studies related to this topic have been conducted by a surgical oncology research group at The Ohio State University [26–30, 34–38, 43, 52]. This group has demonstrated the value of the SVI as a risk stratification tool in the Medicare cancer patient population, especially among those who had underwent resection surgery. Yet, the SVI could also be applied to analyze outcomes in cancer patients in other settings. All studies identified in this review used a retrospective study design, but the SVI may be utilized as a stratification tool for prospective studies. For instance, the SVI could be applied to measure the equity of patient recruitment for studies/trials in terms of SDOH [60].

In addition, the increased availability of more advanced therapeutic agents (e.g., targeted therapy, immune checkpoint inhibitor therapy) is expected to reduce cancer morbidity and mortality [61]. Yet, vulnerable populations may experience disparities in access to potentially life-saving treatment options. Identification of these vulnerable populations using comprehensive data and objective metrics such as the SVI will facilitate societal efforts to improve access to guideline-concordant care and promote health equity. An example of a localized effort to improve cancer care access within a disadvantaged population can be found in an intervention called the Citywide Colon Cancer Control Coalition [62]. In seeking to promote colorectal cancer awareness and increase colonoscopy screening rates in New York City, this intervention was effective in decreasing overall colorectal cancer incidence, but colorectal cancer incidence and mortality rates remained disproportionally high among non-Hispanic Blacks compared to non-Hispanic Whites, Hispanics, and Asians for over ten years. Furthermore, borough-level analyses revealed that colorectal cancer mortality in the boroughs of Staten Island and the Bronx—boroughs with a higher proportion of Black residents—were significantly higher than those in the boroughs of Queens and Manhattan. Future healthcare policy informed by neighborhood-level characteristics can aid policymaking groups in identifying vulnerable communities from the outset that could benefit from further interventions and resources.

The SVI, which has demonstrated its utility in supporting vulnerable populations during national disaster responses, could therefore be used to guide future endeavors to reduce excess cancer morbidity and mortality in specific communities. In one validation study, Carmichael et al. applied various indices of neighborhood-level disadvantage, namely the Community Needs Index [8], Area Deprivation Index [9], Distressed Communities Index [10], and SVI, to the same dataset and demonstrated that the SVI performs similarly to the other indices [63]. A key advantage to using the SVI is the ability to stratify patient outcomes by specific social variables or subthemes, as listed in Fig. 1 [34, 63]. The SVI can thus be used as a key index not only for research but also for policymaking groups with access to data at the census tract-level. Furthermore, in recognition of innovative applications of the SVI to health disparity research, the CDC has newly developed an extended SVI with two additional subthemes on healthcare access/infrastructure and medical vulnerability [64]. This new tool, called the Minority Health SVI, also enhanced the minority status subtheme by expanding race and ethnicity and language variables. The Minority Health SVI has recently been applied to COVID-19 research, where it has demonstrated a positive association between medical vulnerability and COVID-19 incidence and mortality [16]. Future oncology research could apply the Minority Heath SVI to identify racial and ethnic minority communities with disproportionate vulnerability to adverse outcomes [64].

### Limitations

As with most population-based studies, the inferences drawn at the group, community, or population level may not be applicable at the individual patient level. The reliability of the SVI tool may also require further validation using individual-level SES data. In addition, while this review used a systematic method to search multiple databases and screen results in order to reduce selection bias, relevant articles not yet published, posted, or indexed into the queried databases at the time of the search may have been missed. For articles that used the SVI to examine a patient population not explicitly identified as at risk for cancer or diagnosed with cancer, we reviewed the supplemental materials and all references cited in the methods before deciding whether to include or exclude. Nevertheless, some relevant articles may have been inadvertently excluded during the full-text review stage. This review may be subject to publication bias in which studies with statistically significant findings are more likely to be published or presented. By including gray literature resources such as conference abstracts in the search process, we sought to reduce this bias in order to enhance the comprehensiveness of this review.

## Conclusion

In summarizing the current literature as related to the use of SVI in oncology research, this review highlights the SVI as a promising tool for examining health disparities in cancer patients. The results of this study demonstrate the wide-ranging applications of the SVI to examining geographic disparities in potentially cancer-causing exposures, cancer diagnoses, cancer treatments, and cancer mortality as well as in post-operative care, survivorship care, and end-of-life care among cancer patients. Since the SVI dataset is geocoded, it may be linked with other geocoded datasets to generate actionable findings. By highlighting disparities in health-related outcomes and identifying discrete geographic areas with increased risk, the SVI could inform the development and implementation of geographically targeted interventions to decrease cancer morbidity and mortality at the community level. For instance, future research may employ the SVI to investigate disparities in access to cancer screening interventions and diagnostic procedures for early detection. The results from these studies may guide the regional dissemination of public health campaigns and educational programs designed to reduce the burden of cancer.

## Supplementary Information

Below is the link to the electronic supplementary material.

Supplementary file1 (DOCX 90 kb)

Supplementary file2 (DOCX 22 kb)

Supplementary file3 (DOCX 27 kb)

Supplementary file4 (DOCX 57 kb)

Supplementary file5 (DOCX 59 kb)

Supplementary file6 (DOCX 94 kb)

## Data Availability

All data compiled in this review have been made available to the reader.

## Abbreviations

SDOH: Social determinants of health
SES: Socioeconomic status
SVI: Social vulnerability index
CDC: Centers for disease control and prevention
ACS: American Community Survey
COVID-19: Coronavirus disease 2019
PRISMA: Preferred Reporting Items for Systematic Reviews and Meta-Analyses
STROBE: Strengthening the Reporting of Observational Studies in Epidemiology
CMS: Centers for Medicare and Medicaid Services
SEER: Surveillance, Epidemiology, and End Results Program
GEOID: Geographic identifier
