# Supplementary material for: The social vulnerability index as a risk stratification tool for health disparity research in cancer patients: a scoping review
Source: Cancer Causes Control. 2023 Apr 7;34(5):407–20. doi: 10.1007/s10552-023-01683-1 (PMC10080510; doi:10.1007/s10552-023-01683-1)
Supplement: Supplementary file 1 — Supplementary file1 (DOCX 90 kb) [file 10552_2023_1683_MOESM1_ESM.docx]

**Supplementary Figure S1.** PRISMA flow diagram of the study selection process

**
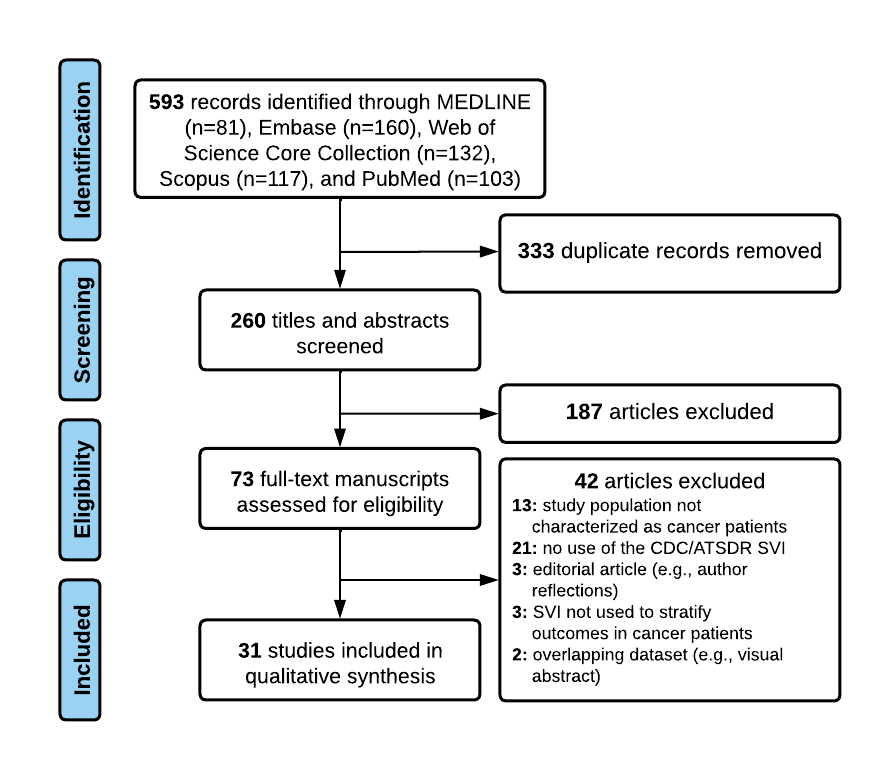
**

**Abbreviations:** PRISMA, Preferred Reporting Items for Systematic Reviews and Meta-Analyses; Centers for Disease Control and Prevention; ATSDR, Agency for Toxic Substances and Disease Registry; SVI, social vulnerability index

**Reference:** Moher D, Liberati A, Tetzlaff J, Altman DG. Preferred reporting items for systematic reviews and meta-analyses: the PRISMA statement. *BMJ*. 2009;339:b2535
