# Supplementary material for: The social vulnerability index as a risk stratification tool for health disparity research in cancer patients: a scoping review
Source: Cancer Causes Control. 2023 Apr 7;34(5):407–20. doi: 10.1007/s10552-023-01683-1 (PMC10080510; doi:10.1007/s10552-023-01683-1)
Supplement: Supplementary file 2 — Supplementary file2 (DOCX 22 kb) [file 10552_2023_1683_MOESM2_ESM.docx]

## **Supplementary Methods S1.** Search strategies for **(A)** MEDLINE (Ovid), **(B)** Embase (Ovid), **(C)** Web of Science Core Collection (Clarivate), **(D)** Scopus (Elsevier), and **(E)** PubMed (National Library of Medicine) for relevant English-language articles published from database inception to November 22, 2021

**Search Strategies**

**A.** Database(s): **Ovid MEDLINE(R) ALL**1946 to November 22, 2021

| **#** | **Searches** | **Results** |
| --- | --- | --- |
| 1 | exp Neoplasms/ | 3574503 |
| 2 | Early Detection of Cancer/ | 31072 |
| 3 | (cancer* or carcinoma* or neoplas* or tumo?r* or leuk?emia* or lymphoma* or melanoma* or sarcoma* or adenocarcinoma* or mesotheliom* or surgery or surgical).ti,ab,kf. | 5365752 |
| 4 | or/1-3 [Cancer] | 6114909 |
| 5 | ((social or county) adj3 vulnerability).ti,ab,kf. | 1527 |
| 6 | 4 and 5 [Cancer + SVI] | 78 |
| 7 | limit 6 to english language | 68 |

**B.** Database(s): **Embase Classic+Embase**1947 to 2021 November 22

| **#** | **Searches** | **Results** |
| --- | --- | --- |
| 1 | exp Neoplasm/ | 5303902 |
| 2 | Early Detection of Cancer/ | 7731 |
| 3 | (cancer* or carcinoma* or neoplas* or tumo?r* or leuk?emia* or lymphoma* or melanoma* or sarcoma* or adenocarcinoma* or mesotheliom* or surgery or surgical).ti,ab,kw. | 7308464 |
| 4 | or/1-3 [Cancer] | 8326676 |
| 5 | ((social or county) adj3 vulnerability).ti,ab,kw. | 1665 |
| 6 | 4 and 5 [Cancer + SVI] | 140 |
| 7 | limit 6 to english language | 133 |

## **C.** Database(s): **Scopus**

( TITLE-ABS-KEY ( cancer* OR carcinoma* OR neoplas* OR tumo?r* OR leuk?emia* OR lymphoma* OR melanoma* OR sarcoma* OR adenocarcinoma* OR mesotheliom* OR surgery OR surgical ) ) AND ( TITLE-ABS-KEY ( ( social OR county ) PRE/3 vulnerability ) ) AND ( LIMIT-TO ( LANGUAGE , "English" ) )

## **D.** Database(s): **Web of Science Core Collection**

TS=(cancer* or carcinoma* or neoplas* or tumo?r* or leuk?emia* or lymphoma* or melanoma* or sarcoma* or adenocarcinoma* or mesotheliom* or surgery or surgical) AND TS=((social or county) NEAR/3 vulnerability)

Refined by: LANGUAGES: ( ENGLISH )

Timespan: All years. Indexes: SCI-EXPANDED, SSCI, A&HCI, CPCI-S, CPCI-SSH, BKCI-S, BKCI-SSH, ESCI, CCR-EXPANDED, IC.

## **E.** Database(s): **PubMed**

(("Neoplasms"[MeSH Terms] OR "Early Detection of Cancer"[MeSH Terms] OR "cancer*"[Text Word] OR "carcinoma*"[Text Word] OR "neoplas*"[Text Word] OR "tumor*"[Text Word] OR "tumour*"[Text Word] OR "leukemia*"[Text Word] OR "leukaemia*"[Text Word] OR "lymphoma*"[Text Word] OR "melanoma*"[Text Word] OR "sarcoma*"[Text Word] OR "adenocarcinoma*"[Text Word] OR "mesotheliom*"[Text Word] OR "surgery"[Text Word] OR "surgical"[Text Word]) AND ("social vulnerability"[Text Word] OR (("counties"[All Fields] OR "county"[All Fields] OR "county s"[All Fields]) AND "vulnerability"[Text Word]))) AND (english[Filter])
