# Supplementary material for: The social vulnerability index as a risk stratification tool for health disparity research in cancer patients: a scoping review
Source: Cancer Causes Control. 2023 Apr 7;34(5):407–20. doi: 10.1007/s10552-023-01683-1 (PMC10080510; doi:10.1007/s10552-023-01683-1)
Supplement: Supplementary file 3 — Supplementary file3 (DOCX 27 kb) [file 10552_2023_1683_MOESM3_ESM.docx]

**STROBE Checklist for Observational Studies: Scoring Worksheet**

| **Title** |  | | |
| --- | --- | --- | --- |
| **1^st^ Author** |  | **Study PI Affiliation** |  |
| **Year** |  | **Manuscript No.** |  |
| **Journal** |  | **Reviewer Initials** |  |

| **Section** | **No.** | **Criteria** | **Complete (1 point)** | **Incomplete (0.5 points)** | **Absent**  **(0 points)** | **Comments** |
| --- | --- | --- | --- | --- | --- | --- |
| **Title & Abstract** |  |  |  |  |  |  |
| Summary | **1** | *(a)* Indicate the study’s design  *(b)* Provide an informative summary of what was done and what was found |  |  |  |  |
| **Introduction** |  |  |  |  |  |  |
| Background/  rationale | **2** | Explain the scientific background and rationale for the investigation being reported |  |  |  |  |
| Objectives | **3** | State specific objectives (including any prespecified hypotheses) |  |  |  |  |
| **Methods** |  |  |  |  |  |  |
| Study design | **4** | Present key elements of study design *(OK if in Introduction)* |  |  |  |  |
| Setting | **5** | Describe the setting, locations, and relevant dates* (including periods of recruitment, exposure, follow-up, and data collection) |  |  |  |  |
| Participants | **6** | *Cross-sectional study:* Provide the eligibility criteria, and report the sources and methods of selection of participants  *For other study designs, please refer to the STROBE statement.* |  |  |  |  |
| Variables | **7** | *(a)* Clearly define all outcomes, exposures, predictors, potential confounders, and effect modifiers  *(b)* Give diagnostic criteria, if applicable |  |  |  |  |
| Data sources/  measurement | **8** | *(a)* For each variable of interest, give sources of data and details of methods of assessment (measurement)  *(b)* Describe comparability of assessment methods (if >1 group) |  |  |  |  |
| Bias | **9** | Describe any efforts to address potential sources of bias |  |  |  |  |
| Study size | **10** | Explain how the study size was arrived at |  |  |  |  |
| Quantitative variables | **11** | *(a)* Explain how quantitative variables were handled in the analyses  *(b)* Describe which groupings were chosen (if applicable) and why |  |  |  |  |
| Statistical methods | **12** | *(a)* Describe all statistical methods, including those used to control for confounding  *(b)* Describe any methods used to examine subgroups and interactions  *(c)* Explain how missing data were addressed  *(d)* Describe analytical methods taking account of sampling strategy (if applicable)  *(e)* Describe any sensitivity analyses |  |  |  |  |
| **Results** |  |  |  |  |  |  |
| Participants | **13** | Report numbers of individuals at each stage of study |  |  |  |  |
| Descriptive data | **14** | *(a)* Give characteristics of study participants (e.g., demographic, clinical, social) and information on exposures and potential confounders  *(b)* Indicate number of participants with missing data for each variable of interest |  |  |  |  |
| Outcome data | **15** | Report numbers of outcome events or summary measures |  |  |  |  |
| Main results | **16** | *(a)* Give unadjusted estimates and, if applicable, confounder-adjusted estimates and their precision (e.g., 95% confidence interval)  *(b)* Make clear which confounders were adjusted for and why they were included  *(c)* Report category boundaries when continuous variables were categorized |  |  |  |  |
| Other analyses | **17** | Report other analyses done (e.g., analyses of subgroups and interactions and sensitivity analyses) |  |  |  |  |
| **Discussion** |  |  |  |  |  |  |
| Key results | **18** | Summarize key results with reference to study objectives |  |  |  |  |
| Limitations | **19** | Discuss limitations of the study, taking into account sources of potential bias or imprecisions |  |  |  |  |
| Interpretation | **20** | Give a cautious overall interpretation of the results considering objectives, limitations, multiplicity of analyses, results from similar studies, and other relevant evidence |  |  |  |  |
| Generalizability | **21** | Discuss the generalizability (external validity) of the results |  |  |  |  |
| **Other Information** | |  |  |  |  |  |
| Funding | **22** | Report the source of funding and the roles of funders for the present study |  |  |  |  |
| **Total** |  |  |  |  |  | **STROBE Score: ___** |

**Complete (1 point):** All relevant criteria are present.

**Incomplete (0.5 points):** Some of the relevant criteria are present. Please list missing features under Comments.

**Absent (0 points):** None of the relevant criteria are present.

** For studies based on disease registries or databases, many of the checklist items may not be applicable, e.g., the dates of recruitment, numbers eligible at each stage of the study, reasons for nonparticipation, or flow diagrams.*

**STROBE statement:** von Elm E, Altman DG, Egger M, Pocock SJ, Gøtzsche PC, Vandenbroucke JP; STROBE Initiative. Strengthening the reporting of observational studies in epidemiology (STROBE) statement: guidelines for reporting observational studies. *Br Med J*. 2007 Oct 20;335(7624):806-8.

**STROBE worksheet adapted from:** Adams AD, Benner RS, Riggs TW, Chescheir NC. Use of the STROBE checklist to evaluate the reporting quality of observational research in obstetrics. *Obstet Gynecol*. 2018;132(2):507-512.
