# Supplementary material for: The social vulnerability index as a risk stratification tool for health disparity research in cancer patients: a scoping review
Source: Cancer Causes Control. 2023 Apr 7;34(5):407–20. doi: 10.1007/s10552-023-01683-1 (PMC10080510; doi:10.1007/s10552-023-01683-1)
Supplement: Supplementary file 4 — Supplementary file4 (DOCX 57 kb) [file 10552_2023_1683_MOESM4_ESM.docx]

**Supplementary Table S1.** Inclusion and exclusion criteria for study eligibility

|  | **Inclusion Criteria** | **Exclusion Criteria** |
| --- | --- | --- |
| **Articles** | - Articles published in the English language - Any study designs | - Articles published in a language other than English - Editorial articles (e.g., perspectives, opinions, commentaries) - Duplicate article or overlapping dataset |
| **Populations** | - Patients identified as at-risk for cancer; or - Patients diagnosed with cancer; or - Patients undergoing a therapeutic intervention for a cancer diagnosis; or - Patients receiving cancer survivorship care such as longitudinal surveillance for cancer recurrence | - Patient populations characterized by medical condition(s) other than cancer |
| **SDOH Measures** | - Use of the CDC/ATSDR SVI to analyze patient outcomes | - No use of the CDC/ATSDR SVI to analyze patient outcomes |
| **Outcome Measures** | - Any | - None reported |

**Abbreviations:** SDOH, social determinants of health; CDC, Centers for Disease Control and Prevention; ATSDR, Agency for Toxic Substances and Disease Registry; SVI, social vulnerability index
