# Supplementary material for: The social vulnerability index as a risk stratification tool for health disparity research in cancer patients: a scoping review
Source: Cancer Causes Control. 2023 Apr 7;34(5):407–20. doi: 10.1007/s10552-023-01683-1 (PMC10080510; doi:10.1007/s10552-023-01683-1)
Supplement: Supplementary file 5 — Supplementary file5 (DOCX 59 kb) [file 10552_2023_1683_MOESM5_ESM.docx]

**Supplementary Table S2.** Characteristics of included articles and their primary and secondary outcomes as related to SVI

| **Article^Ref^**, Study Design, Quality, *Cancer Care Continuum* | **Cancer Type(s)** | **Research Question** | **Data Sources** | **Patient Population** | **Primary Outcomes** | **Primary Effect Measures** | **Selected Secondary Outcomes** | **Selected Secondary Effect Measures** |
| --- | --- | --- | --- | --- | --- | --- | --- | --- |
| **Abbas et al., 2021, *Ann Surg Oncol*^26^**  C-S (R)  Excellent quality  *End-of-life* | Colon  Lung  Pancreatic  Rectal  Esophageal | Are county-level SVI and race/ethnicity associated with patterns of hospice utilization?  *Hos*p*ice utilization*: early, ≥28 d prior to death; late, ≤3 d prior to death | Medicare, 2013-2017 | Patients who underwent a resection for colon (n=26,287), lung (n=16,645), pancreatic (n=6,183), rectal (n=3,174), or esophageal (n=1,427) cancer and who lived ≥30 d after surgery and died; ≥65 y.o. | Overall hospice utilization relative to SVI (per +10%) × race/ethnicity | SVI × Minority vs. SVI × White, OR 0.97 (95% CI 0.96-0.99), p<0.05*  *Inter*p*retation*: incremental reduction in the probability of hospice utilization among minority patients as SVI increased by 10% | Early and late hospice utilization relative to SVI (per +10%) × race/ethnicity | *Early*: SVI × Minority vs. SVI × White, OR 0.94 (95% CI 0.91-0.97), p<0.05*  *Late*: SVI × Minority vs. SVI × White, OR 0.97 (95% CI 0.94-1.01), p>0.05 |
| **Azap et al., 2020, *Surgery*^27^**  C-S (R)  Excellent quality  *Treatment Recovery* | Pancreatic  Liver | Is county-level SVI associated with the probability of post-op textbook outcomes?  *Textbook outcome*: absence of post-op complications, prolonged LOS, 90-day readmission, or 90-day mortality | *Patient data*: Medicare, 2013-2017  *Hos*p*ital data*: CMS Hospital General Information; CMS Final Rule; Leapfrog | Patients who had pancreatic (n=18,841) or liver resection (n=13,301) for cancer at 11 cancer centers and 54 NCI-CC-affiliated hospitals; ≥65 y.o. | Post-op textbook outcomes relative to SVI (quartiles, high vs. low) | *Pancreas*: OR 0.89 (95% CI 0.82-0.97), p<0.05*  *Liver*: OR 0.89 (95% CI 0.80-0.98), p<0.05* | Medicare expenditures per admission relative to SVI (quartiles, high vs. low) | *Pancreas*: OR 1.05 (95% CI 1.02-1.09), p<0.05*  *Liver*: OR 1.04 (95% CI 1.00-1.08), p<0.05* |
| **Azap et al., 2021, *Ann Surg Oncol*^28^**  C-S (R)  Excellent quality  *Treatment* | Pancreatic/ PDAC, early-stage | Are county-level SVI and race/ethnicity associated with the probability of undergoing resection or receiving chemotherapy? | SEER-Medicare, 2004-2016 | Older patients with early-stage PDAC (n=15,931); 71-82 y.o. | PDAC resection and chemotherapy receipt relative to SVI (quartiles; per +10%) | *PDAC resection*:  Low SVI, 38.0% vs. Average SVI, 34.3% vs. High SVI, 31.9%; all, p<0.001**  Per +10% in SVI, OR 0.98 (95% CI 0.97-1.00), p=0.052  *Neoadjuvant chemo*: Per +10% in SVI, OR 0.98 (95% CI 0.97-0.99), p=0.045*  *Adjuvant chemo*: Per +10% in SVI, OR 0.99 (95% CI 0.97-1.00), p=0.054 | PDAC resection and chemotherapy receipt relative to SVI (quartiles, high vs. low) × race | *PDAC resection:*  Minority, OR 0.87 (95% CI 0.66-1.15), p=0.32  White, OR 0.97 (95% CI 0.86-1.10), p=0.64  *Neoadjuvant chemo*:  Minority, OR 0.62 (95% CI 0.52-0.73), p<0.001**  White, OR 0.97 (95% 0.86-1.10), p=0.63  *Adjuvant chemo*:  Minority, OR 0.1.09 (95% CI 0.57-2.08), p=0.79  White, OR 0.72 (95% CI 0.54-0.96), p=0.02* |
| **Azap et al., 2021, *JAMA Surg*^29^**  C-S (R)  Excellent quality  *Treatment* | Liver: HCC, early-stage (stage I/II) | Are county-level SVI and race/ethnicity associated with the probability of undergoing resection or transplant? | SEER-Medicare, 2004-2017 | Patients with early-stage HCC (n=10,888); ≥65 y.o. | Liver resection or transplant relative to SVI (quartiles, high vs. low) | OR 1.18 (95% CI 1.01-1.38), p<0.05* | Liver resection or transplant relative to SVI (quartiles, high vs. low) × race | *Black & Hispanic:* OR 1.41 (95% CI 1.10-1.81), p<0.05*  *White:* OR 0.99 (95% CI 0.82-1.19), p>0.05 |
| **Barmash et al., 2020, *J Am Coll Surg*^30^**  C-S (R)  Low quality  *Treatment*  *Treatment Recovery* | Colon | Is county-level SVI associated with emergent vs. non-emergent colon resection and post-op outcomes? | Medicare, 2016-2017 | Patients who underwent a colon resection for colon cancer (n~35,324), diverticulitis (n~15,154), or IBD (n~2,323); ≥65 y.o. | Non-elective colon resection relative to SVI (per +20%) | *Cancer*: +1.4% per +20% in SVI (95% CI +1.0%-1.7%), p<0.05*  *Diverticulitis*: +0.9% per +20% in SVI (95% CI +0.4%-1.4%), p<0.05*  *IBD*: +2.4% per +20% in SVI (95% CI +1.0%-3.7%), p<0.05* | Post-op complications, 30-day mortality, and 30-day readmission for non-elective vs. elective colon resection | Post-op complications, 53.9% vs. 16.5%, p<0.05*  30-day mortality, 12.5% vs. 2.5%, p<0.05*  30-day readmission, 7.7% vs. 5.1%, p<0.05* |
| **Bhandari et al., 2021, *Blood*^31^**  1-cohort (R)  Low quality  N/A | AML  Other liquid tumors, unspeci-fied | Is census tract-level SVI associated with 1-year non-relapse mortality? | Single institution in California, 2013-2019 | Patients who underwent a 1^st^ allogenic HCT (n=1,602)  652 (40.7%) with AML | 1-year non-relapse mortality relative to SVI (overall; tertiles, high vs. average & low) | Overall SVI: negative trend on multivariable analysis, coefficient 0.0056 (95% CI 0.001-0.010), p=0.017*  High SVI vs. Average SVI & Low SVI: HR 1.41 (95% CI 1.08-1.83), p=0.011* | 1-year non-relapse mortality relative to census tract-level SVI subthemes (tertiles, high vs. average & low) | *SES*: HR 1.43 (95% CI 1.10-1.86), p=0.008**  *HC&D*: HR 1.32 (95% CI 1.02-1.71), p=0.036*  *MS&L*: HR 1.40 (95% CI 1.08-1.83), p=0.013*  *HT&T*: p>0.05 |
| **Bowers et al., 2020, *Lab Invest*^32^**  C-S (R)  Low quality  *Diagnosis* | Leukemia | Does leukemia subtype incidence vary within and between areas of SES disparity? | SEER-21, 2000-2016 | Patients with leukemia (77 subtypes) across 727 counties in 14 states; no. patients NR | Age-adjusted leukemia subtype incidence relative to Gini coefficient and income inequality ratio | *No. subty*p*es with significant associations*: Gini coefficient, 37; income inequality ratio, 38 | Age-adjusted leukemia subtype incidence relative to county-level SVI (quartiles), residential segregation, and rurality | *No. subty*p*es with significant associations*: SVI, 31; residential segregation, 31; rurality, 24 |
| **Carmichael et al., 2022, *Am J Surg*^33^**  C-S (R)  Good quality  *Treatment Recovery* | Colon  Rectal | Is census tract-level SVI associated with increased risk of post-op 30-day morbidity following colectomy? | 5 hospitals within a large academic health system in Colorado including local patients in ACS-NSQIP, 2012-2017 | Patients who underwent a colectomy for colorectal cancer (n=392), diverticular disease (n=272), or other reasons (n=312) | Post-op mortality relative to SVI (quartiles; high vs. others), unadjusted | OR 1.84 (95% CI 1.35-2.52), p<0.001** | Post-op mortality relative to SVI (quartiles; high vs. others), adjusted | OR 1.37 (95% CI 0.95-1.98), p=0.10 |
| **Dalmacy et al., 2021, *Surgery*^34^**  C-S (R)  Excellent quality  *Treatment*  *Treatment Recovery* | Pancreatic  Liver | Is county-level SVI associated with risk of fragmented post-op care?  *Fragmented* p*ost-o*p *care (FPC)*: 90-day readmission at a different hospital | Medicare, 2013-2017 | Patients who underwent pancreatic resection (n=8,053) or liver resection (n=3,089) and had ≥1 readmission within 90 d; ≥65 y.o.  6,216 (55.8%) with cancer (% liver and % pancreatic NR) | Non-FPC vs. FPC by SVI (overall; subthemes) | *Overall SVI*: Non-FPC, 51.3% (95% CI 27.9%-69.4%) vs. FPC, 52.5% (95% CI 29.3%-70.4%); p=0.026*  *SES*: p=0.002**  *HC&D*: p<0.001**  *MS&L*: p<0.001**  *HT&T*: p=0.34 | FPC relative to SVI (overall, high vs. low; subthemes, high vs. low) | *Overall SVI*: OR 1.14 (95% CI 1.01-1.30), p=0.042*  *SES*: p<0.001**  *HC&D*: p<0.001**  *MS&L*: p<0.001**  *HT&T*: p=0.98 |
| **Diaz et al., 2021, *Ann Surg Oncol*^35^**  C-S (R)  Excellent quality  *Treatment* | Lung  Rectal  Pancreatic  Esophageal | Is county-level SVI associated with utilization of high-volume hospitals for high-risk cancer operations? | *Patient data*: California OSHPD, 2012-2016  *Hos*p*ital data*: Leapfrog | Patients who underwent a high-risk cancer operation for lung (n=14,403), rectal (n=7,520) pancreatic (n=3,744), or esophageal (n=1,270) cancer | Cancer resection at a high-volume hospital relative to SVI (overall) | *Lung*: OR 0.67 (95% CI 0.51-0.88), p<0.05*  *Rectal*: OR 0.76 (95% CI 0.58-0.98), p<0.05*  *Pancreatic*: OR 0.61 (95% CI 0.44-0.84), p<0.05*  *Eso*p*hageal*: OR 0.39 (95% CI 0.24-0.65), p<0.05* | Risk-adjusted travel time to destination hospital relative to SVI (quartiles; high vs. others) | *Lung*: +20.51 min (95% CI +15.91–25.11), p<0.05*  *Rectal*: +25.04 min (95% CI +19.89–30.18), p<0.05*  *Pancreatic*: +44.97 min (95% CI +37.24–52.71), p<0.05*  *Eso*p*hageal*: +41.54 min (95% CI +29.56-53.52), p<0.05* |
| **Diaz et al., 2021, *J Gastrointest Surg*^36^**  C-S (R)  Excellent quality  *Treatment*  *Treatment Recovery* | Colon | Is county-level SVI associated with the probability of having a non-elective vs. elective colon resection? | MedPAR, 2016-2017 | Patients who underwent colon resection for diverticulitis (n=11,812) or colon cancer (n=33,312); 65-99 y.o. | Non-elective colon resection rates for cancer or diverticulitis relative to SVI (quartiles) | *Cancer*: Low SVI, 26.6% (95% CI 25.7%-27.5%) vs. High SVI, 29.4% (95% CI: 28.4%-30.3%), p<0.05*  *Diverticulitis*: Low SVI, 37.1% (95% CI 35.7%-38.5%) vs. High SVI, 40.5% (95% CI: 39.0%-42.0%), p<0.05* | Post-op complications, 30-day mortality, 30-day readmission, and Medicare expenditures in non-elective colon resections relative to SVI (quartiles, high vs. low)  *Outcomes for elective colon resections also re*p*orted in this article* | *Post-o*p *com*p*lications*: overall, p=0.044*; cancer, p=0.016*; diverticulitis, p=0.819  *30-day mortality*: overall, p=0.253; cancer, p=0.052; diverticulitis, p=0.221  *30-day readmission*: overall, p=0.552, cancer, p=0.757; diverticulitis, p=0.614  *Ex*p*enditures*: overall, p=0.023*; cancer, p=0.004**; diverticulitis, p=0.713 |
| **Diaz et al., 2021, *J Surg Oncol*^37^**  C-S (R)  Excellent quality  *Treatment*  *Treatment Recovery* | Lung  Colon | What is the impact of county-level SVI and racial/ethnic residential diversity on post-op outcomes? | Medicare, 2016-2017 | Patients who underwent resection for lung (n=33,803) or colon (n=21,939) cancer; ≥65 y.o. | 30-day mortality relative to SVI (quartiles, high vs. low) and SVI × residential diversity (quartiles) | High SVI vs. Low SVI, OR 1.02 (95% CI 1.01-1.03), p<0.05*  Low SVI × High diversity, 3.2% (95% CI 3.0-3.5) vs. High SVI × Low diversity, 5.2% (95% CI 4.6-5.8), p<0.05* | Post-op complications, serious complications, 30-day mortality, and 30-day readmission relative to SVI (quartiles, high vs. low) | Post-op complications, OR 0.84 (95% CI 0.77-0.91), p<0.001**  Serious complications, OR 0.84 (95% CI 0.76-0.93), p=0.001**  30-day mortality, OR 0.75 (95% CI 0.64-0.87), p<0.001*  30-day readmission, OR 1.03 (95% CI 0.94-1.14), p=0.486 |
| **Diaz et al., 2021, *Surgery*^38^**  C-S (R)  Excellent quality  *Treatment*  *Treatment Recovery* | Pancreatic  Liver | Is county-level SVI associated with use of a high-volume or Magnet recognition hospital? | Medicare, 2013-2017 | Patients who underwent resection for pancreatic (n=13,393) or liver (n=3,594) cancer; 65-99 y.o. | Cancer operation at a high-volume or Magnet recognition hospital relative to SVI (quartiles, high vs. low) | *High-volume hos*p*ital*: OR 0.98 (95% CI 0.97-0.99), p<0.005*  *Magnet recognition hos*p*ital*: OR 1.03 (95% CI 1.01-1.04), p=0.002** | Post-op complications and 30-day mortality at low- vs. high-volume hospitals relative to SVI (quartiles) | *Post-o*p *com*p*lications*: Low SVI, 26.6% vs. 20.5%; Average SVI, 30.7% vs 24.0%; High SVI, 32.7% vs. 25.8%; all, p<0.05*  *30-day mortality*: Low SVI, 5.3% vs. 3.0%; Average SVI, 6.5% vs. 3.7%; High SVI: 7.8% vs 4.4% all, p<0.05* |
| **Ganatra et al., 2021, *Circulation*^39^**  C-S (R)  Low quality  N/A | Any | Is county-level SVI associated with mortality from CVD and concomitant cancer (cardio-oncology)? | WONDER, 2014-2018 | Patients with CVD and concomitant cancer; no. patients NR | AAMR due to concomitant cancer and CVD relative to SVI (quartiles) | Higher AAMR in areas with high SVI | CVD mortality and cancer-related mortality relative to SVI (quartiles) | Higher CVD mortality in areas with high SVI  Higher cancer-related mortality in areas with high SVI |
| **Grant et al., 2021, *Blood*^40^**  C-S (R)  Low quality  N/A | Liquid tumors (e.g., MM, leukemia, lympho-ma) | Is county-level SVI associated with physical frailty? | Carolina Senior Registry, NR  Registry for Adults with Plasma Cell Disorders, NR | Patients in North Carolina with hematologic malignancy (n=338) | Frailty relative to SVI (overall and subthemes) | *Overall*: 73% vs. 45%, p=0.01*  *SES*: 68% vs. 47%, p=0.005**  *HT&T*: 66% vs. 54%, p=0.48 | Baseline demographics (e.g., race, education) relative to SVI | *Race*: p<0.001**  *Education*: p<0.001** |
| **Grant et al., 2021, *J Clin Oncol*^41^**  C-S (R)  Low quality  *Treatment* | MM | Is county-level SVI associated with MM trial availability in North Carolina? | ClinicalTrials.gov, accessed on Jan. 24, 2021 | Registered MM trials in North Carolina (456 trials); no. patients NR | MM trials relative to SVI (per +1%) | –3.3% in trial incidence per +1% in SVI; IRR 0.97, p=0.008** | MM trials relative to racial/ethnic residential diversity | More diverse counties vs. less diverse counties, IRR 1.01, p=0.08 |
| **Hawley et al., 2022, *JAMA Netw Open*^42^**  1-cohort (R)  Excellent quality  *Treatment* | Any, except NMSCs | Is there a spatiotemporal association in COVID-19 outcomes? | COVID-19 and Cancer Consortium (CCC19), Mar.–Nov. 2020 | Patients with cancer and laboratory-confirmed SARS-CoV-2 infection with follow-up data (n=4,749), ≥18 y.o. | 30-day all-cause mortality relative to SVI (per +10%) | OR 1.04 (95% CI 0.95-1.13) | Composite outcome (mechanical ventilation, ICU admission, all-cause mortality) relative to SVI (per +10%) | OR 0.98 (95% CI 0.87-1.09 |
| **Hyer et al., 2021, *J Am Coll Surg*^43^**  C-S (R)  Excellent quality  *Treatment*  *Treatment Recovery* | Colon  Lung  Rectal  Esophageal | Are there differences in textbook outcomes relative to county-level SVI and race/ethnicity?  *Textbook outcomes*: absence of post-op complications, prolonged LOS, 90-day readmission, and 90-day mortality | Medicare, 2013-2017 | Patients with colon (n=113,929), lung (n=70,642), rectal (n=14,849), or esophageal (n=4,380) cancer who underwent a cancer operation; ≥65 y.o. | Textbook outcomes relative to SVI (quartiles) | Low SVI, 57.9% vs. Average SVI, 56.1% vs. High SVI, 54.4%; p<0.001**  High SVI vs. Low SVI, OR 0.83 (95% CI 0.78-0.87), p<0.001** | Textbook outcomes relative to SVI (quartiles, high vs. low) × race | *Minority*: OR 0.78 (95% CI 0.71-0.85), p<0.05*  *White*: OR 0.90 (95% CI 0.87-.93), p<0.05* |
| **Labiner et al., 2022, *J Gastrointest Surg*^44^**  C-S (R)  Good quality  *Treatment Recovery* | Pancreas?  Liver? | Does county-level SVI subtheme analysis in patients undergoing hepatopancreatic surgery better stratify potential gaps in identifying risks of post-op complications? | Medicare, 2013-2017 | Patients who underwent pancreatic resection or liver resection for any indication (n=37,707)  26,540 (70.4%) with cancer | Textbook outcome by SVI profile  *Textbook outcome*: absence of post-op complications, extended LOS, 90-day readmission, or 90-day mortality | *High vulnerability* (SVI profiles 4 vs. 5): OR 0.89 (95% CI 0.83-0.95), p<0.05*  *Average vulnerability* (SVI profiles 2 vs. 3): OR 1.13 (95% CI 1.04-1.23), p<0.05* | 90-day mortality by SVI profile | *High vulnerability* (SVI profiles 4 vs. 5): OR 1.29 (95% CI 1.15-1.44), p<0.05*  *Average vulnerability* (SVI profiles 2 vs. 3): OR 1.14 (95% CI 1.01-1.30), p<0.05* |
| **McAlarnen et al., 2021, G*ynecol Oncol*^45^**  C-S (R)  Good quality  *Survivorship Care* | Ovarian  Uterine  Cervical  Vulval/ vaginal | Are gynecologic malignancies and census tract-level SVI associated with the utilization of virtual visits? | Single institution in New York City, 2020 (6-month period at beginning of COVID-19 pandemic) | Patients who had a virtual visit with gynecology oncology (n=270 females) | Utilization of phone vs. video virtual visits by SVI (median) | Phone, median SVI 23% (IQR 12%-40%) vs. Video, median SVI 25% (IQR 9%-52%), p=0.5 | Disease site, stage, and race/ethnicity by SVI (median) | Different disease sites, p=0.5  Difference disease stages, p=0.5  Different races/ethnicities, p<0.001** |
| **McAlarnen et al., 2022, *Cancer E***p***idemiol Biomark Prev*^46^**  1-cohort (R)  Low quality  *Diagnosis* | Cervical | Are demographic and geographic factors (e.g., census tract-level SVI) associated with diagnosis of locally advanced cervical cancer? | Single institution in Milwaukee, Wisconsin, 2016-2021 | Patients with locally advanced cervical cancer who received brachytherapy (n=66) | Race/ethnicity relative to median SVI (overall) | Asian, 0.96/1 vs. African American, 0.96/1 vs. Hispanic 0.53/1 vs. American Indian or Alaska Native, 0.30/1 vs. White, 0.29/1; p=0.005** | Race/ethnicity relative to median SVI (subthemes) | *SES*: Asian > others; p<0.05*  *HC&D*: African American > others; p<0.05*  *MS&L*: Asian > others; p<0.05*  *HT&T*: p>0.05 |
| **Mock et al., 2021, *Transplant Cell Ther*^47^**  C-S (R)  Excellent quality  *Treatment* | AML | Do HCT rates for AML vary between different regions in Virginia? | *Patient data*: Virginia Cancer Registry, 2013-2017  *AML trans*p*lant data*: CIBMTR | Patients in Virginia diagnosed with AML (n=818) | HCT within 2 years of diagnosis by geographic region | *Region with ≥25% Black* p*o*p*ulation*: OR 0.58 (95% CI 0.38-0.89)  *Region with higher % of* p*o*p*ulation with college education*: OR 1.02 (95% CI 1.00-1.03) | HCT within 2 years of AML diagnosis relative to census tract-level SVI (overall) | OR 0.37 (95% CI 0.166-0.824), p=0.015* |
| **Pan et al., 2021, *Hepatology*^48^**  C-S (R)  Low quality  *Prevention*  *Diagnosis* | Liver: HCC | Do neighborhood-level (e.g., census tract-level) characteristics impact alcohol-associated liver disease outcomes (including development of HCC)? | Large academic medical center and associated community hospital in New York City, 2012-2019 | Patients with (1) advanced alcohol-associated liver disease or (2) advanced liver disease with alcohol use disorder (total, n=1,286)  99 (7.7%) developed HCC | HCC diagnosis (HCC vs. no HCC) relative to SVI (overall), race/ethnicity, and SDOH measures | SVI, p=0.1  Race/ethnicity, p=0.03*  Poverty rate, p=0.07  Median household income, p<0.001**  % limited English speakers, p=0.17  % foreign-born, p=0.46  % less than high school education, p<0.001** | Mortality (died vs. alive) relative to SVI (overall), race/ethnicity, and SDOH measures | SVI, p=0.52  Race/ethnicity, p=0.92  Poverty rate, p=0.19  Median household income, p=0.008**  % limited English speakers, p=0.47  % foreign-born, p=0.40  % less than high school education, p=0.64 |
| **Papageorge et al., 2021, *J Am Coll Surg*^49^**  C-S (R)  Excellent quality  *Diagnosis* | Liver: HCC | Was county-level SVI associated with the impact of Medicaid expansion on the diagnosis of HCC? | SEER, 2010-2016 | Patients with HCC (n=19,751) from 12 states; <65 y.o.  Identified in 18 registries (~28% U.S. population) | Pre- vs. post- expansion HCC diagnosis (early- vs. late-stage) in expansion vs. non-expansion states | p=0.4280 | Pre- vs. post-expansion HCC diagnosis (early- vs. late-stage) relative to state expansion status and SVI (specific variables) | *Ex*p*ansion states*:  Poverty, p=0.5251  Limited English, p=0.3398  No vehicle, p=0.2528  *Non-ex*p*ansion states*:  Poverty, p=0.0686  Limited English, p=0.1948  No vehicle, p=0.1382 |
| **Parks et al., 2022, *JAMA*^50^**  C-S (R)  Excellent quality  *Prevention* | Any | Is county-level tropical cyclone exposure associated with county-level cause-specific mortality (including cancer-specific mortality)? | *Patient data*: National Center for Health Statistics, 1988-2018  *Wind data*: Parameter-elevation Regres-sions on Indepen-dent Slopes Model | Patients who died from 1 of 6 causes (injury, infectious & parasitic disease, respiratory disease, CVD, neuropsychiatric condition, or cancer) and lived in a county that experienced at least 1 cyclone during the study period (n=33,619,393 deaths in 1,206 counties) | % change in death rates in the following 6 months per 1-day increase in monthly tropical cyclone or hurricane-only exposures | *Cancer-s*p*ecific:* -0.3% (95% CI -0.9%-+0.3%), p>0.05  *Outcomes for other causes of death also re*p*orted this article* | Additional no. deaths per 1,000,000 for 2018 monthly age-standardized median rate | *Cancer-s*p*ecific*: 100.4 to 100.1 deaths; -0.3 days (95% CI -0.9-+0.3); p>0.05  *Outcomes for other causes of death also re*p*orted this article* |
| **Puvvula et al., 2021, *Water*^51^**  C-S (R)  Good quality  *Prevention*  *Diagnosis* | All pediatric cancers except NMSCs | Are atrazine concentrations at watersheds associated with the incidence of pediatric cancers in Nebraska? | *Patient data*: Nebraska Cancer Registry, 1987-2016  *Atrazine data*:  Nebraska Clearing-house, STORET, USGS  *Watershed data*: USGS-National Hydro-graphy | Pediatric patients with cancer (n=2,559); ≤19 y.o. at time of diagnosis | Pediatric cancer incidence relative to atrazine concentrations in surface and groundwater samples from watersheds, unadjusted | Positive association between pediatric cancer and atrazine concentrations in watersheds with low, medium, and high atrazine vs. watersheds with negligible atrazine | Pediatric cancer incidence relative to atrazine concentrations in surface and groundwater samples from watersheds, adjusted for census tract-level SVI (overall) | Positive association between pediatric cancer and atrazine concentrations in watersheds with low, medium, and high atrazine vs. with negligible atrazine |
| **Rice et al., 2021, *Ann Surg Oncol*^52^**  C-S (R)  Excellent quality  *End-of-life* | Liver: HCC | What factors (e.g., county-level SVI) are associated with hospice utilization and healthcare expenditures at the end-of-life? | SEER-Medicare, 2004-2016 | Patients with HCC who died of HCC and had claims in their last year of life (n=14,369) | Hospice utilization relative to SVI (quartiles; continuous) and race/ethnicity | Low SVI vs. High SVI, OR 1.12 (95% CI 0.98-1.38), p=0.07  Average SVI vs. High SVI, OR 0.74 (95% CI 0.65-0.85), p<0.001**  *Minority*: positive association between early hospice utilization and SVI | Healthcare expenditures (inpatient and outpatient) relative to hospice utilization | *In*p*atient*: Hospice $7,900 (IQR $0-$26,000) vs. No Hospice, $18,000 (IQR $400-$49,100), p<0.001**  *Out*p*atient*: Hospice $900 (IQR $0-$4,500) vs. No Hospice, $2,200 (IQR $200-$7,900), p<0.001** |
| **Taylor et al., 2021, *Gastroenterology*^53^**  C-S (R)  Low quality  *Treatment* | Colon | Is Medicaid-Medicare dual eligibility status associated with non-elective surgery for colon cancer and variation in outcomes and spending? | Medicare, 2014-2018 | Fee-for-service patients who underwent non-elective resection surgery (not diversion alone) for colon cancer (n=146,041); ≥65 y.o. | Non-elective resection surgery between patients with Medicaid-Medicare vs. Medicare alone | OR 1.52 (95% CI 1.47-1.57), p<0.001** | Non-elective resection surgery between patients with Medicaid-Medicare vs. Medicare alone by county-level SVI (quartile?) | p>0.05 |
| **Ying et al., 2020, *Hepatology*^54^**  C-S (R)  Low quality  *Prevention*  *Diagnosis* | Liver: HCC | Are racial and neighborhood-level factors (e.g., SVI) associated with adverse outcomes in those with cirrhosis due to viral hepatitis? | Large academic medical center and associated community hospital in New York City, 2012-2019 | Patients with cirrhosis due to viral hepatitis (n=359)  143 (39.8%) with HCC | HCC diagnosis (HCC vs. no HCC) relative to SVI (quartiles, area level NR), race/ethnicity, and SDOH measures | SVI, p=0.16  Race/ethnicity, p=0.10  Poverty rate, p=0.28  Median household income, p=0.004**  % limited English speakers, p=0.04*  % foreign-born, p=0.37  % less than high school education, p=0.03* | Mortality (died vs. alive) relative to SVI (quartiles, area level NR), race/ethnicity, and SDOH measures | SVI, p=0.02*  Race/ethnicity, NR  Poverty rate, p>0.05  Median household income, p>0.05  % limited English speakers, p>0.05  % foreign-born, p>0.05  % less than high school education, p>0.05 |
| **Ying et al., 2021, *Hepatology*^55^**  C-S (R)  Low quality  *Prevention*  *Diagnosis* | Liver: HCC, advanced-stage (T2+) | Is SVI associated with advanced-stage HCC diagnosis in those with cirrhosis due to viral hepatitis? | Tertiary care hospital and associated community hospital in New York City, 2012-2020 | Patients with cirrhosis due to viral hepatitis who were admitted (n=348), excluding those with liver transplants prior to admission  98 (28.2%) with advanced-stage HCC | Advanced-stage HCC diagnosis or 5-year survival relative to SVI (data level NR, area level NR) | Advanced-stage HCC vs. No HCC, p=0.48  5-year survival, p>0.05 | Advanced-stage HCC diagnosis (advanced-stage HCC vs. no HCC) relative to race and other SODH measures | Race/ethnicity, p=0.01*  % limited English speakers, p=0.01*  % foreign-born, p=0.01*  % less than high school education, p=0.03* |
| **Zhang et al., 2022, *Health Aff (Project Hope)*^56^**  C-S (R)  Excellent quality  *Treatment* | Colon | Is county-level SVI associated with unplanned surgeries for 1 of 3 specified access-sensitive conditions (colectomy for colon cancer, AAA repair, or incisional hernia repair)? | MedPAR, 2014-2018 | Fee-for-service patients undergoing surgery for 1 of 3 access-sensitive conditions (n=292,966); 65-99 y.o.  203,732 (29.4%) underwent colectomy for colon cancer | Risk-adjusted rate of unplanned surgery relative to SVI (quintile, high vs. low) | *Colectomy*: OR 1.12, p<0.001***  *AAA re*p*air*: OR 1.07, p<0.001***  *Incisional hernia re*p*air*: OR 1.11, p<0.001*** | Risk-adjusted rate of post-op outcomes relative to SVI (quintile, high vs. low) | *30-day mortality*: OR 0.96, p<0.001***  *Any com*p*lications*: OR 0.99, p<0.10  *Serious com*p*lications*: OR 0.99, p>0.10  *Re-o*p*erations*: OR 1.01, p<0.10  *Readmissions*: OR 1.00, p>0.10 |

* denotes p<0.05; ** denotes p<0.01; × denotes interaction effect; NR, not reported

**Abbreviations (in alphabetical order):**

1-cohort, single-cohort

AAMR, age-adjusted mortality ratio

ACS-NSQIP, American College of Surgeons National Surgical Quality Improvement Program

AML, acute myeloid leukemia

CI, confidence interval

CIBMTR, Center for International Blood and Marrow Transplant Research

CMS, Centers for Medicare and Medicaid Services

COVID-19, coronavirus disease 2019

C-S, cross-sectional

CVD, cardiovascular disease

HC&D, Household Composition & Disability (SVI Subtheme 2)

HCC, hepatocellular carcinoma (liver cancer)

HCT, hematopoietic stem cell transplantation

HR, hazard ratio

HT&T, Housing Type & Transportation (SVI Subtheme 4)

IBD, inflammatory bowel disease

IQR, interquartile range

IRR, incidence rate ratio

LOS, length of stay

MedPAR, Medicare Provider Analysis and Review

MM, multiple myeloma

MS&L, Minority Status & Language (SVI Subtheme 3)

NCI-CC, National Cancer Institute-designated Cancer Center

NMSC, non-melanoma skin cancer

OR, odds ratio

OSHPD, Office of State-wide Health Planning and Development, a department within the California Health and Human Services Agency

PDAC, pancreatic ductal adenocarcinoma (pancreatic cancer)

(R), retrospective

SDOH, social determinants of health

SEER, Surveillance, Epidemiology, and End Results Program, managed by the National Cancer Institute

SES, socioeconomic status (also SVI Subtheme 1)

STORET, STOrage and RETrieval database, managed by the Environmental Protection Agency

SVI, social vulnerability index, developed by the Centers for Disease Control and Prevention

TNM, Tumor, Node, Metastasis

USGS, U.S. Geological Survey

WONDER, Wide-ranging Online Data for Epidemiological Research, managed by the CDC
