# Supplementary material for: The social vulnerability index as a risk stratification tool for health disparity research in cancer patients: a scoping review
Source: Cancer Causes Control. 2023 Apr 7;34(5):407–20. doi: 10.1007/s10552-023-01683-1 (PMC10080510; doi:10.1007/s10552-023-01683-1)
Supplement: Supplementary file 6 — Supplementary file6 (DOCX 94 kb) [file 10552_2023_1683_MOESM6_ESM.docx]

**Supplementary Table S3.** Distribution of included articles along the cancer care continuum plus mortality outcomes, non-applicable articles not shown

| **Article^Ref^** (Author, Year, *Journal*) | **Cancer Etiology**  **& Prevention** | **Early**  **Detection** | **Cancer Diagnosis** | **Cancer Treatment** | **Treatment Recovery** | **Survivor-**  **ship Care** | **End-of-Life** | **Mortality**  **Outcomes** |
| --- | --- | --- | --- | --- | --- | --- | --- | --- |
| Abbas et al., 2021, *Ann Surg Oncol*^26^ |  |  |  |  |  |  | Hospice |  |
| Azap et al., 2020, *Surgery*^27^ |  |  |  |  | Post-op complications, HC expenditures |  |  | Post-op 90-day |
| Azap et al., 2021, *Ann Surg Oncol*^28^ |  |  |  | Resection, chemotherapy |  |  |  |  |
| Azap et al., 2021, *JAMA Surg*^29^ |  |  |  | Resection, solid organ transplant |  |  |  |  |
| Barmash et al., 2020, *J Am Coll Surg*^30^ |  |  |  | Resection | Post-op complications |  |  | Post-op 30-day |
| Bhandari et al., 2021, *Blood*^31^ |  |  |  |  |  |  |  | 1-year non-relapse |
| Bowers et al., 2020, *Mod Pathol*^32^ | Neighborhood* |  | All-stage |  |  |  |  |  |
| Carmichael et al., 2022, *Am J Surg*^33^ |  |  |  |  | Post-op complications |  |  | Post-op 30-day |
| Dalmacy et al., 2021, *Surgery*^34^ |  |  |  | Resection | Post-op care |  |  |  |
| Diaz et al., 2021, *Ann Surg Oncol*^35^ |  |  |  | Resection |  |  |  |  |
| Diaz et al., 2021, *J Gastrointest Surg*^36^ |  |  |  | Resection | Post-op complications, HC expenditures |  |  | Post-op 30-day |
| Diaz et al., 2021, *J Surg Oncol*^37^ |  |  |  | Resection | Post-op outcomes |  |  |  |
| Diaz et al., 2021, *Surgery*^38^ |  |  |  | Resection | Post-op outcomes |  |  |  |
| Ganatra et al., 2021, *Circulation*^39^ |  |  |  |  |  |  |  | Cancer-related |
| Grant et al., 2021, *J Clin Oncol*^41^ |  |  |  | Clinical trial |  |  |  |  |
| Hawley et al., 2022, *JAMA Netw Open*^42^ | Neighborhood* |  |  | Anticancer therapy |  |  |  | 30-day all-cause |
| Hyer et al., 2021, *J Am Coll Surg*^43^ |  |  |  | Resection surgery | Post-op complications |  |  | Post-op 90-day |
| Labiner et al., 2022, *J Gastrointest Surg*^44^ |  |  |  |  | Post-op complications |  |  | Post-op 90-day |
| McAlarnen et al., 2021, G*ynecol Oncol*^45^ | Neighborhood* |  |  |  |  | Virtual visits |  |  |
| McAlarnen et al., 2022, *Cancer Epidemiol*  *Biomark Prev*^46^ |  |  | Locally advanced |  |  |  |  |  |
| Mock et al., 2021, *Transplant Cell Ther*^47^ | Neighborhood* |  |  | Stem cell transplant |  |  |  |  |
| Pan et al., 2021, *Hepatology*^48^ | Substance use, neighborhood* |  | All-stage |  |  |  |  | All-cause |
| Papageorge et al., 2021, *J Am Coll Surg*^49^ | Neighborhood* |  | Early-stage, late-stage |  |  |  |  |  |
| Parks et al., 2022, *JAMA*^50^ | Environmental characteristics |  |  |  |  |  |  | Cancer-specific |
| Puvvula et al., 2021, *Water*^51^ | Environmental contaminants,  neighborhood* |  | All-stage |  |  |  |  |  |
| Rice et al., 2021, *Ann Surg Oncol*^52^ |  |  |  |  |  |  | Hospice, HC expenditures |  |
| Taylor et al., 2021, *Gastroenterology*^53^ |  |  |  | Resection |  |  |  |  |
| Ying et al., 2020, *Hepatology*^54^ | Oncogenic virus, neighborhood* |  | All-stage |  |  |  |  | All-cause |
| Ying et al., 2021, *Hepatology*^55^ | Oncogenic virus, neighborhood* |  | Late-stage |  |  |  |  | All-cause |
| Zhang et al., 2022, *Health Aff (Project Hope)*^56^ |  |  |  | Resection |  |  |  |  |

***** neighborhood (area-level) characteristics as opposed to patient (individual-level) characteristics, in addition to the SVI

**Abbreviations:** post-op, post-operative; HC, healthcare; SVI, social vulnerability index
